# Artificial Intelligence-Based Diagnostic Support System for Patent Ductus Arteriosus in Premature Infants

**DOI:** 10.3390/jcm13072089

**Published:** 2024-04-03

**Authors:** Seoyeon Park, Junhyung Moon, Hoseon Eun, Jin-Hyuk Hong, Kyoungwoo Lee

**Affiliations:** 1 Department of Computer Science, Yonsei University, 50 Yonsei-ro, Seoul 03722, Republic of Korea; seoyeon.park@yonsei.ac.kr (S.P.); kyoungwoo.lee@yonsei.ac.kr (K.L.); 2 Department of Pediatrics, Yonsei University College of Medicine, 50-1 Yonsei-ro, Seoul 03722, Republic of Korea; hseun@yuhs.ac; 3 School of Integrated Technology, Gwangju Institute of Science and Technology, 123 Cheomdangwagi-ro, Gwangju 61005, Republic of Korea; jh7.hong@gist.ac.kr

**Keywords:** patent ductus arteriosus, premature infant, diagnostic support system, electronic health record, machine learning

## Abstract

**Background**: Patent ductus arteriosus (PDA) is a prevalent congenital heart defect in premature infants, associated with significant morbidity and mortality. Accurate and timely diagnosis of PDA is crucial, given the vulnerability of this population. **Methods**: We introduce an artificial intelligence (AI)-based PDA diagnostic support system designed to assist medical professionals in diagnosing PDA in premature infants. This study utilized electronic health record (EHR) data from 409 premature infants spanning a decade at Severance Children’s Hospital. Our system integrates a data viewer, data analyzer, and AI-based diagnosis supporter, facilitating comprehensive data presentation, analysis, and early symptom detection. **Results**: The system’s performance was evaluated through diagnostic tests involving medical professionals. This early detection model achieved an accuracy rate of up to 84%, enabling detection up to 3.3 days in advance. In diagnostic tests, medical professionals using the system with the AI-based diagnosis supporter outperformed those using the system without the supporter. **Conclusions**: Our AI-based PDA diagnostic support system offers a comprehensive solution for medical professionals to accurately diagnose PDA in a timely manner in premature infants. The collaborative integration of medical expertise and technological innovation demonstrated in this study underscores the potential of AI-driven tools in advancing neonatal diagnosis and care.

## 1. Introduction

Premature infants face a multitude of health challenges, among which patent ductus arteriosus (PDA) is particularly significant. PDA is a congenital heart defect characterized by the persistence of the ductus arteriosus after birth [1]. This condition predominantly affects premature infants, substantially increasing morbidity and mortality risks [2,3]. Given their clinical vulnerability, premature infants with PDA require specialized care [4]. Despite advancements in medical treatments enhancing survival rates, managing PDA remains critical to mitigating the risk of long-term disabilities [5,6].

The timely and accurate diagnosis of PDA is crucial for the effective health management of premature infants [7,8]. Echocardiography, a traditional heart examination method using ultrasound, provides precise results but often requires resources and expertise not universally available, especially in resource-constrained settings [9,10,11]. Consequently, without advanced diagnostic tools, many hospitals rely solely on clinical signs in the initial days following birth. This reliance may delay diagnosis and lead to suboptimal treatments [7,12].

To address these diagnostic challenges, we developed a PDA diagnostic support system designed to aid medical professionals in the prompt and accurate diagnosis of PDA. Utilizing the vast amount of patient data available in electronic health record (EHR) systems, our system offers a comprehensive solution to neonatal care. It integrates data visualization and machine learning techniques for efficient interpretation of patient information, comprising a data viewer, data analyzer, and an artificial intelligence (AI)-based diagnosis supporter. These components work in concert to facilitate intuitive data visualization, in-depth analysis, and early symptom detection, all the while ensuring user-friendliness and seamless integration into medical workflows.

We evaluated the effectiveness of our PDA diagnostic support system through a retrospective study involving EHR data from 409 premature infants admitted to Severance Children’s Hospital. Our designed diagnostic test and an exit survey with medical professionals assessed the system’s performance. The diagnostic test results underscore the system’s ability to enable early and accurate diagnoses, and the feedback from the exit survey further highlights the system’s applicability in clinical settings. The key contributions of this study include:

- Developing a comprehensive PDA diagnostic support system incorporating a data viewer, data analyzer, and AI-based diagnosis supporter, tailored to meet the needs of medical professionals;
- Processing and utilizing diverse types of EHR data from 409 premature infants for designing the PDA diagnostic support system;
- Conducting diagnostic tests with medical professionals to empirically validate the system’s effectiveness, demonstrating its potential to improve diagnostic accuracy.

## 2. Related Work

Recent advancements in AI have significantly improved medical diagnostics and patient care. AI techniques have become increasingly prominent in the medical domain, assisting clinicians in making precise and timely decisions that enhance patient outcomes [13]. However, despite numerous studies focusing on adult diseases, research on pediatric conditions, especially those affecting neonates, remains relatively limited [14,15,16].

The field of predictive research in neonatal care has attracted considerable attention due to the profound impact that neonatal conditions can have on childhood mortality rates [17,18]. Various studies have explored AI-based approaches within neonatal care, utilizing patient data from EHR systems in neonatal intensive care units (NICUs) [19,20,21,22,23]. For instance, Batista et al. [20] demonstrated the effectiveness of machine learning models in predicting severe diseases and assessing infant mortality risks using physiological data from NICU settings.

Further investigations have concentrated on specific severe diseases prevalent among premature infants. Jiang et al. [24] applied diverse machine learning techniques to predict respiratory distress syndrome, a chronic neonatal lung condition, using clinical data. López-Martínez et al. [25] and Masino et al. [26] developed early detection models for sepsis in infants, drawing on insights from physiological data in NICU records. Additionally, Na et al. [27] made attempts to predict PDA using machine learning techniques, although their approach, relying mainly on static factors, lacked the flexibility to accommodate the evolving conditions of individual patients, limiting its clinical applicability.

While the potential of AI to revolutionize neonatal care is clear, its widespread adoption in healthcare settings has been hampered by challenges. A significant barrier is the perceived complexity and lack of transparency in several AI models, causing reluctance among medical professionals [28]. Trust in these tools can be fostered through enhanced transparency and interpretability [29]. To address this need, clinical decision support systems (CDSSs) have emerged to bridge this gap. These systems not only provide diagnostic outcomes but also explain their underlying reasoning, meeting the needs of medical professionals [30]. Significant advancements have been made in this field. For example, Massafra et al. [31] proposed a CDSS for personalized breast cancer treatment plans, integrating feature importance techniques to enhance model interpretability. Zhou et al. [32] developed a CDSS for hypertension management using a knowledge graph to improve medication decision support. This system not only simplifies knowledge management but also provides highly visual and interpretable recommendations, aiming to enhance guideline compliance and prescription efficacy in clinical practices. Nevertheless, the neonatal domain, particularly concerning conditions like PDA in premature infants, is still awaiting comprehensive CDSS solutions.

## 3. Materials and Methods

### 3.1. Preliminary Survey

Prior to the development of our PDA diagnostic support system, we conducted a preliminary survey with 20 medical professionals from the Department of Pediatrics at Severance Children’s Hospital, labeled P1 to P20. The survey aimed to identify essential functions for the system and gather insights for creating a practical diagnostic tool, comprising four parts.

In the first part, we collected demographic data from the participants, including age, gender, occupational roles, and years of medical service. This approach, aligned with the methodology used in Ozok’s work [33], ensured a comprehensive understanding of the participants’ roles and backgrounds. This demographic information is critical for subsequent statistical analyses and understanding variations by professional role.

The second part explored the current strategies employed by medical professionals for diagnosing PDA, utilizing a mix of multiple-choice and open-ended questions. This part aimed to identify key factors and methods in PDA assessment, providing valuable insights into existing diagnostic practices, essential elements considered significant in diagnosis, and challenges encountered. These insights informed the design and optimization of our diagnostic support system to ensure its alignment with clinical practices.

The third part focused on the collection of patient information during medical consultations to identify inconveniences and potential areas for improvement. Through multiple-choice and short-answer questions, we assessed the sources of patient information used in diagnosis and treatment and explored the integration of these sources into medical decision-making. Insights from this part helped to align patient data sources with clinical needs, aiding in the development of a diagnostic support system that addresses real-world challenges. Varied response formats allowed participants to provide specific details from their experiences and observations.

In the fourth part, we investigated medical professionals’ perspectives on incorporating AI into healthcare, identifying areas that could benefit from AI support. Participants rated their satisfaction with the current EHR system on a five-point Likert scale, covering aspects such as user-friendliness, data integration, and performance. We also inquired whether an AI-powered system for predicting PDA risk would be beneficial, probing for reasons and preferred features of such AI system. Drawing on Zamora et al.’s work [34] for a reliable assessment, this part captured perspectives on AI integration in healthcare and areas needing improvement.

Based on the survey results, we identified key requirements for our PDA diagnostic support system. Firstly, the system needs to **be universally accessible**, catering to the varying diagnostic skills of medical professionals. Secondly, it should **incorporate a broad spectrum of indicators**, including vital signs and prenatal factors, for effective detection of PDA. Thirdly, the system should **emphasize user-friendliness and offer an informative data presentation**, considering the prevalent reliance on the EHR system by medical professionals, despite their expressed dissatisfaction with its interface. Lastly, the system should **incorporate AI-driven functionalities**, as there was a pronounced interest among medical professionals in leveraging AI to assist treatment decisions and provide comprehensive patient insights.

### 3.2. EHR Dataset of Premature Infants

This section describes the EHR dataset of premature infants and outlines the pre-processing steps undertaken. This dataset served as a crucial resource in developing our PDA diagnostic support system, as informed by insights from the preliminary survey.

#### 3.2.1. Description

The dataset spans a decade, from 1 January 2009 to 31 December 2018, comprising medical records of 409 premature infants admitted to the NICUs at Severance Children’s Hospital in Seoul, Republic of Korea. These records represent a valuable collection for studying PDA diagnosis in premature infants and were securely maintained within the hospital’s EHR system. Initially, the dataset contained records of 436 infants; however, we excluded 27 records of infants who unfortunately passed away within the first three days after birth to mitigate early mortality biases.

Responses from the preliminary survey (Part 3) indicated a primary reliance on EHR data, including critical echocardiography results, for diagnosing PDA. Thus, this dataset played an essential role in the development of our PDA diagnostic support system. The gestational age of the infants in the study ranged from 23 to 37 weeks (mean: 28.9, STD: 2.9), with birth weights between 380 to 1490 g (mean: 1094.2, STD: 278.5). This study was approved by the Severance Institutional Review Board (4-2019-0504).

The dataset covers the period from pregnancy to 15 days after birth, incorporating factors that could influence PDA diagnosis. The focus on the first 15 days after birth stemmed from its acknowledgment by the medical community as a critical time period for PDA symptom manifestation. We divided the study population of 409 premature infants into two groups: the symptomatic PDA group (132 infants receiving pharmacological or surgical treatment within the first 15 days after birth) and the asymptomatic PDA group (277 infants without such interventions). Statistical analysis was performed using Student’s *t*-test for continuous variables, presented as mean ± standard deviation, and the chi-squared test for categorical variables, presented as percentages. These analyses aimed to identify significant differences between the groups, as detailed in Table 1.

**Figure 1:**
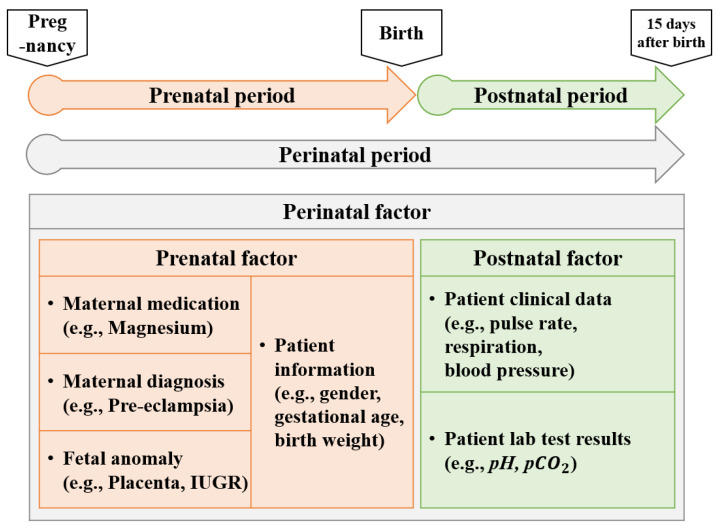
Dataset categorization. Comprehensive maternal and patient data are included from pregnancy to the initial 15 days after birth (IUGR: intrauterine growth restriction).

Furthermore, we categorized the dataset based on the time of recording, distinguishing between prenatal and postnatal factors to identify crucial times for diagnosis. This approach met the needs of medical professionals in identifying factors that influence PDA symptoms at different stages. This categorization, illustrated in Figure 1, includes three categories: prenatal, postnatal, and perinatal factors. Prenatal factors include maternal particulars, such as antenatal hospital visits, and patient information at birth, like birth weight. In contrast, postnatal factors include data recorded from one hour after birth throughout the hospitalization, encompassing clinical data and laboratory test results. Perinatal factors merge both prenatal and postnatal factors, offering a comprehensive view from before birth to hospitalization. This methodical approach allows for a thorough consideration of all factors potentially impacting PDA diagnosis in premature infants.

#### 3.2.2. Pre-Processing

In the pre-processing step, our objective was to standardize heterogeneous data types present in our dataset, including descriptive, numerical, and event sequence data, into formats suitable for both data visualization and machine learning analysis. The descriptive data, often found in an unstructured form due to the variability in documentation practices over time, were transformed into binary variables based on the presence or absence of specific records, such as medication types, diagnoses, and echocardiography findings. This transformation process adheres to established machine learning methodologies in healthcare applications [26], making it possible for the AI model to effectively process critical information.

In addition to descriptive data, our dataset comprised numerical data, which were subject to normalization. This steps ensures that numerical values are scaled to a standardized range, facilitating the AI model’s ability to analyze these data points without bias towards any particular scale of values. For event sequence data, which mirror the dynamic nature of patient conditions, we employed a strategy to extract eight daily representative values (minimum, maximum, median, average, percentile (25th/75th), standard deviation, and variance) for each patient, following the method shown by Baig et al. [35]. This approach was designed to accommodate variations in measurement frequencies, counts, and intervals, attributable to differing patient conditions and medical practice standards. The selection of features from these event sequences aimed to facilitate the early detection of changes indicative of PDA symptom development after birth [36].

Through this pre-processing, we extracted 19 features from prenatal factors and 80 features from postnatal factors, ensuring a robust dataset for subsequent analysis. This process not only standardizes the diverse types of data within our dataset but also prepares them for effective interpretation and analysis by our AI model, setting a solid foundation for the diagnostic support system we aim to develop.

### 3.3. AI-Based PDA Diagnostic Support System

**Figure 2:**
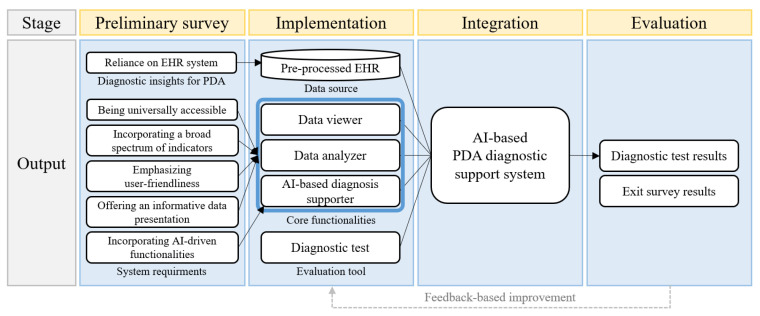
Workflow of the development process for the AI-based PDA diagnostic support system. Diagnostic insights for PDA and system requirements, gathered from the preliminary survey, informed our system’s design and implementation decisions. These components cohesively merge to form our AI-based PDA diagnostic support system. The system’s effectiveness is subsequently evaluated based on diagnostic test results and feedback from an exit survey.

**Figure 3:**
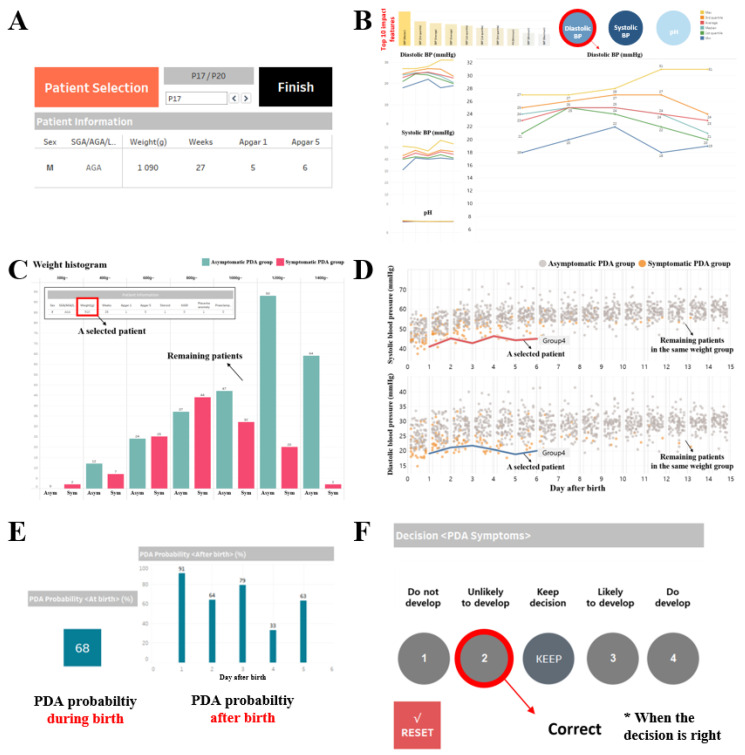
Our designed core functionalities and evaluation tool for the AI-based PDA diagnostic support system. (**A**,**B**): Data viewer; (**C**,**D**): data analyzer; (**E**): AI-based diagnosis supporter; and (**F**): diagnostic test.

Our AI-based PDA diagnostic support system is designed to assist medical professionals in diagnosing PDA in premature infants by integrating data visualization with AI-driven early detection models, thereby enhancing their diagnostic capabilities. As illustrated in Figure 2, the system utilizes pre-processed EHR data as its data source and equips three core functionalities—a data viewer, a data analyzer, and an AI-based diagnosis supporter—in addition to a diagnostic test, which serves as our designed evaluation tool. To develop this system, we employed the data visualization software Tableau 2021.1 (Seattle, WA, USA) [37], which is renowned for its versatile support of data analysis methods, inspired by the approach in Franklin et al. [38]. Users can access patient records through the data viewer, data analyzer, and AI-based diagnosis supporter. They can also conduct diagnoses using the diagnostic test, shown in Figure 3F. To facilitate user interaction, the system is organized within a main dashboard and three auxiliary dashboards, each equipped with control buttons. These buttons enable straightforward navigation between dashboards and ensure the consistent presentation of patient records, optimizing user experience and diagnostic efficiency.

#### 3.3.1. Data Viewer

The data viewer was developed in response to concerns raised by participants in the preliminary survey regarding the data presentation in the EHR system. Based on feedback collected during the survey (Part 4), we identified specific challenges within the existing EHR system. For instance, half of the participants reported difficulties in viewing long-term patient data. Also, 7 out of the 20 participants highlighted the complexity and limited size of the EHR system’s data, making it inconvenient for review. To address these issues, we developed our data viewer, specifically designed to offer a clearer and more efficient way to represent and access long-term patient data. The integration of the data viewer into the system effectively visualizes medical data and facilitates the communication of patient information to users. Visualization, as highlighted by Faiola and Hillier [39], offers significant advantages for data analysis and decision-making in healthcare.

The data viewer provides two types of information. Firstly, it prominently displays the most general information about the current patient, serving as primary indicators for PDA diagnosis. This layout ensures that users can quickly identify the patient’s status at a glance, as illustrated in Figure 3A. Secondly, it provides visualization of the patient’s time series data. Inspired by Ghassemi et al.’s approach [40], our data viewer uses line plots for visualizing these time series data. In Figure 3B, clinical data are transformed into graphical representations, thereby improving clarity. Users can select relevant indices and view specific data types of interest. By presenting selected data alongside related graphs, users can easily compare and assess the patient’s condition. Different colors are employed to differentiate lines representing six representative values: minimum, maximum, median, average, 25th percentile, and 75th percentile values for each date. The graph is organized chronologically by date and divided into eight-hour intervals, enabling users to monitor changes in the patient’s condition over time. This visualization technique is applied to five clinical data and five laboratory test results, corresponding to postnatal factors.

#### 3.3.2. Data Analyzer

The data analyzer was developed to address the need for a flexible EHR system that prioritizes essential diagnostic information. Feedback from the preliminary survey (Part 4) highlighted challenges with the existing EHR system, which overwhelms users with excessive information on one screen. Participants expressed the desire for the EHR system to provide valuable insights beyond raw medical data. For example, P4 mentioned that “*If the system can provide us with notable insights, it will be effective in terms of patient safety as it can diagnose and treat PDA more quickly*”. In consideration of these requirements, our system incorporates the data analyzer, which presents critical data in a concentrated manner and provides unconventional insights to support medical professionals in diagnosing PDA.

The data analyzer utilizes distribution histograms and jitter plots to compare the selected patient’s data with those of other patients exhibiting similar clinical patterns. From prenatal factors, we selected 10 features based on their importance, as determined by survey responses (Part 2). Features such as birth weight and gestational age were chosen for their wide range of values, facilitating data segmentation. We used an approach inspired by a previous study [41], creating histograms at regular intervals to visualize data for all patients. The selected patient’s data is also depicted in these histogram, as shown in Figure 3C. Binary features, such as medication records and echocardiography findings, are split into two ranges and presented as bar plots, following a similar method proposed in another study [42]. This presentation enables clear representation of patient counts within each range, facilitating distribution between the asymptomatic PDA group and the symptomatic PDA group. Thus, this approach assists users in assessing the proximity of the selected patient’s data to either group.

Regarding postnatal factors, blood pressure (BP) data were emphasized due to their strong correlation with PDA, as revealed by survey responses (Part 2). Most participants in the survey ranked BP measurements as the top indicator for diagnosing PDA among all perinatal factors. Similar to our approach for prenatal factors, we divided the entire patient dataset into asymptomatic and symptomatic PDA groups and visualized this division using jitter plots. An illustrative example of the data analyzer employing a patient’s BP data is presented in Figure 3D. This arrangement empowers users to identify patterns in BP fluctuations for each group, helping them recognize to which group patients with similar BP patterns to the selected patient belong. It also enables users to assess specific trends unique to patients within the symptomatic PDA group.

#### 3.3.3. AI-Based Diagnosis Supporter

As a crucial component of our AI-based diagnostic support system, we developed PDA early detection models with the aim of detecting PDA symptoms early. These models were developed based on the expectations expressed by participants in the preliminary survey (Part 4), where many emphasized the need for an AI-based approach to detect PDA in its early stages. For example, P14 noted that “*It would be helpful to make an early treatment plan if there is a high likelihood of PDA developing in the infant*”. P9 also highlighted the potential of AI to “*detect minute areas that are imperceptible to the human eye (usable as a decision support tool)*”. The PDA early detection models provide the possibility of recognizing PDA symptom development by utilizing a combination of various features categorized by their recording time.

Leveraging insights from our preliminary survey (Part 2), we emphasized the crucial role of BP data in PDA diagnosis. To enhance the performance of our PDA early detection models, we conducted feature engineering, specifically focusing on BP data. Premature infants, due to their physiological instability at birth and vital sign fluctuations, require close monitoring [43,44]. While absolute BP measurements are essential, understanding the temporal evolution of these measurements from birth is equally vital [45,46]. Thus, we introduce novel features to capture the rate of change in BP relative to the initial day of birth. We created nine distinct features by using representative values from BP measurements. The creation procedure is as follows: (1) Select representative values (median, 25th percentile, 75th percentile) for systolic BP (SBP) and diastolic BP (DBP) from BP measurements; (2) compute nine differences in SBP and DBP by combining each representative value; (3) calculate the rate of change based on the elapsed day, using the corresponding difference value from the initial day’s sample within each patient’s data. These newly generated BP-related features, combined with the previously extracted features outlined in Table 2, were integrated into the PDA early detection model.

To establish the AI-based diagnosis supporter in our system, we implemented PDA early detection models using diverse feature sets. This resulted in the creation of four distinct PDA early detection models, each integrating specific feature sets as outlined in Table 2. These models are designated as model_prenatal, model_postnatal, model_perinatal, and model_perinatal + bp, with each model utilizing features categorized by their recording time. Model_prenatal exclusively employs prenatal factors to estimate the probability of PDA symptom development during birth. Model_postnatal solely utilizes postnatal factors to validate PDA symptom detection using data recorded after birth, which characterizes patient condition. Model_perinatal combines both prenatal and postnatal factors. Model_perinatal + bp incorporates our novel BP-related features into model_perinatal. The feature set configuration for each model is shown in Table 3. This systematic approach ensures the exploration of various factors and their potential significance in early PDA symptom detection, enhancing the accuracy and robustness of our diagnostic support system.

Daily sampling was conducted for all 409 patients, with 132 in the symptomatic PDA group and 277 in the asymptomatic PDA group. The symptomatic PDA group’s samples included data collected before PDA symptom development, while the asymptomatic PDA group’s samples included data collected up to 15 days after birth. Due to the varying development times of PDA symptoms, the symptomatic PDA group exhibited diverse sample counts. Each sample underwent binary classification, labeled as 0 (Asymptomatic PDA) or 1 (Symptomatic PDA), forming the basis for subsequent analysis and model training.

For model training, we used scikit-learn [47], a machine learning library, and implemented a 5-fold cross-validation approach. The input data for these models included daily samples from individual patients. These models classify each sample into predefined labels, drawing inspiration from existing works [26,48]. The random forest (RF) algorithm was used to compare the detection accuracy of four feature sets, given its robust generalization capacity and suitability for unbalanced datasets [49]. Model performance was gauged using metrics such as accuracy, precision, recall, and false negative rate (FN rate), which is crucial for identifying unforeseen and latent risks in clinical practice [50,51].

To effectively present the outcomes of our PDA early detection models to users, we integrated the AI-based diagnosis supporter into our system. As illustrated in Figure 3E, the AI-based diagnosis supporter offers two types of results to users. The first provides the probability of PDA symptom development during birth, derived from model_prenatal, which uses prenatal factors up until the patient’s birth. The second type shows how this probability evolves over several days after birth, obtained from model_perinatal, which considers both prenatal and postnatal factors for comprehensive assessment. These probabilities are presented as a one-day bar plot, providing users with a clear view of changes over time. This segmentation empowers users to make informed decisions based on the patient’s current condition. In addition, the system seamlessly integrates the data viewer and data analyzer, designed for visualizing and analyzing patient data, with the AI-based diagnosis supporter. This integration provides users with richer information, aiding a more informed decision-making process.

## 4. Results

In this section, we present the findings from three evaluation studies: the performance assessment of the PDA early detection model utilized as an AI-based diagnosis supporter in the PDA diagnostic support system; a diagnostic test aimed at evaluating our system; and an exit survey that captures feedback from medical professionals who completed the diagnostic test.

### 4.1. Performance of PDA Early Detection Models

As previously highlighted, the timely and accurate diagnosis of PDA is important for effective medical interventions. To address this need, we developed PDA early detection models, a critical component of our AI-based PDA diagnostic support system. We provide details of the experimental setup used to evaluate these models. These models were trained and tested on a medical dataset extracted from the EHR system. We used medical records of 409 patients to construct input samples for the models. To account for varying sample sizes per patient, we selected samples from the symptomatic PDA group using data recorded before PDA symptom development. For the asymptomatic PDA group, we considered data recorded within the first 15 days after birth. This approach resulted in a total of 4726 samples. We evaluated the performance of PDA early detection models based on two key dimensions: accuracy and early detection.

We initially examined the impact of factors categorized by recording time on PDA by comparing detection results based on distinct feature sets, as presented in Table 3. We established four PDA early detection models, each employing different combinations of these feature sets. While it was expected that model_prenatal would outperform model_postnatal due to well-established prenatal risk factors for PDA, such as birth weight, gestational age, and Apgar scores [7,8], the results were surprising. Remarkably, model_postnatal, which exclusively considered postnatal factors, achieved an impressive 83% detection accuracy, surpassing even model_perinatal, which combined both prenatal and postnatal factors. This observation suggested that early detection of PDA symptoms can be facilitated not only by known prenatal risk factors but also by postnatal factors. In this context, model_perinatal exhibited the most reasonable performance among the four models, particularly in terms of the lowest FN rate. This improved FN rate underscored the ability of our detection models to effectively manage cases with positive symptoms in diagnoses. Furthermore, model_perinatal + bp achieved the highest detection accuracy of 84%, highlighting the efficacy of our proposed features that capture BP changes in individual patients for PDA symptom detection.

These models provide both classification outcomes and probabilities of classifying individual samples. This allows for the identification of the exact moment when the models detect PDA symptom development based on daily patient data. The analysis focused on symptomatic PDA cases showed that model_perinatal + bp exhibited an average early detection advantage of 3 days, while model_perinatal achieved an even greater advantage of up to 3.3 days. These results highlighted the potential of our models for early PDA symptom detection, emphasizing the significant impact they can have in clinical practice.

### 4.2. Diagnostic Test

To evaluate our PDA diagnostic support system, we conducted a diagnostic test. This test measured the impact of our AI-based diagnosis supporter on diagnostic outcomes. It involved comparing two versions of our system: one without the AI-based diagnosis supporter and one with it integrated. In this test, each system version assessed a series of 10 patient cases. A total of 19 medical professionals specializing in NICU care, including nine clinicians and 10 nurses, sequentially evaluated these cases using both versions. The clinician group consisted of two fellows and seven residents, while the nurses’ group included three physician assistants and seven general nurses.

We analyzed the diagnostic test results obtained from the medical professionals by comparing the outcomes between the two system versions. The primary distinction between these versions was the presence or absence of the AI-based diagnosis supporter (hereafter referred to as an AI-based supporter).

**Figure 4:**
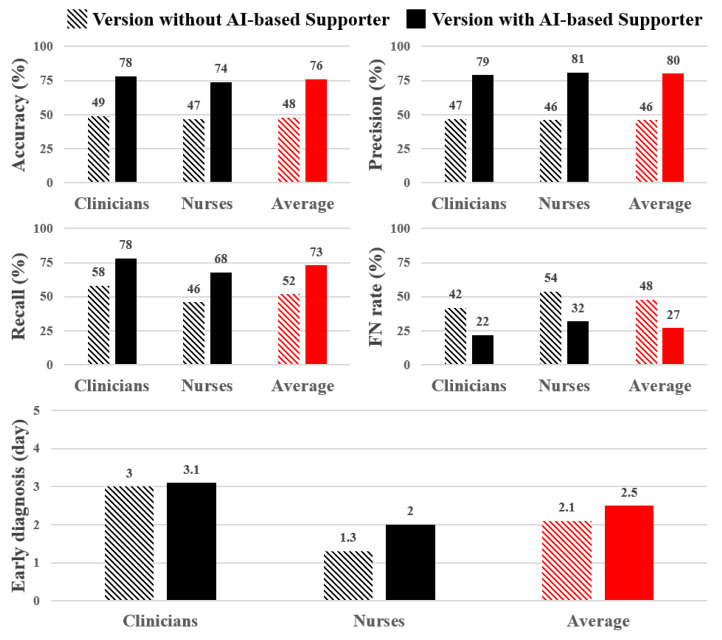
Diagnostic test results for two versions by participant’s medical position. The red color represents the average result across all positions (FN rate: false negative rate).

The results of the diagnostic test were assessed using key metrics such as accuracy, precision, recall, and FN rate. Without the AI-based supporter, participants achieved an average diagnostic accuracy of 48%, as depicted in Figure 4. The precision, recall, and FN rates were found to be 46%, 52%, and 48%, respectively. In contrast, when the AI-based supporter was present in the system, significant improvements in all evaluation metrics were observed. The version with the AI-based supporter exhibited a 28% higher diagnostic accuracy compared to the version without it. Precision and recall were enhanced by 34% and 21%, respectively. Notably, the FN rate was also improved, showing a 21% reduction in the probability of misdiagnosing symptoms compared to the version without the AI-based supporter.

Moreover, the results of the diagnostic test showed that participants could make early diagnoses in both versions (early diagnosis in Figure 4). For cases of symptomatic PDA, participants could make accurate diagnoses on average 2.1 days earlier than the actual development of PDA symptoms in the version without the AI-based supporter. However, with the inclusion of the AI-based supporter, participants demonstrated even earlier diagnostic abilities, making correct decisions 2.5 days prior to the actual development of PDA symptoms. Interestingly, in the clinician group, the impact of the AI-based supporter was relatively modest. Nevertheless, our diagnostic support system still enabled them to achieve early diagnoses on average 3 days earlier than the actual development of PDA symptoms. In the nurse group, participants showed an ability to make early diagnoses, with an average lead time of 1.3 days, even without the AI-based supporter. However, with the AI-based supporter’s assistance, their early diagnostic abilities were further enhanced, allowing them to achieve an average lead time of up to 2 days before the actual development of PDA symptoms.

The diagnostic test, conducted in collaboration with medical professionals, served as robust validation of the efficacy of our proposed system. It highlighted the significant positive impact of the AI-based diagnosis supporter on the accuracy and timeliness of PDA diagnoses, underscoring the practical value of our diagnostic support system in clinical settings.

### 4.3. Exit Survey

To gather valuable insights into users’ experiences, we conducted an exit survey following the diagnostic test completion. The survey aimed to assess overall satisfaction with the system’s functionality and usability. It consisted of eight questions, divided into two categories: system performance and system usability, based on well-established evaluation criteria [34]. Each question employed a five-point Likert scale, ranging from 1 (strongly dissatisfied) to 5 (strongly satisfied). Additionally, we included one question asking which component of the PDA diagnostic support system was the most useful for decision-making.

**Figure 5:**
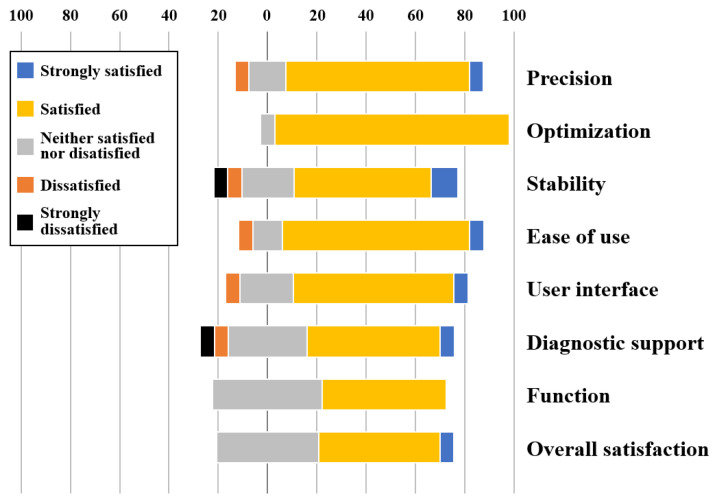
Exit survey results. Precision, Optimization, and Stability are items for evaluating the performance of the system, and the other five items are for evaluating the usability of the system.

Summarized results, as depicted in Figure 5, indicate that participants generally hold a positive view of the system. Particularly noteworthy are the consistently high satisfaction levels reported in terms of system optimization and ease of use. These findings underscored the system’s success in delivering a convenient and user-friendly experience. Conversely, the aspect of system stability received relatively lower satisfaction scores. We recognize this as an area requiring prioritized attention in future work to enhance the system’s reliability and robustness. Lastly, participants highlighted the AI-based diagnosis supporter as the most useful component, emphasizing the value of the symptom development probability it provides for enhancing diagnostic accuracy and clinical decision-making.

## 5. Discussion

While our study has made significant strides towards the development of a PDA diagnostic support system for medical professionals, certain limitations must be addressed for a better understanding of our findings. First, relying on data from a single medical center over a decade may introduce biases due to variations in clinical processes and treatment approaches among different institutions, potentially limiting the system’s applicability to broader healthcare settings. Moreover, the relatively small dataset of 409 patient cases presents challenges for implementing advanced techniques like deep learning, which did not yield substantial improvements compared to machine learning-based models. Future efforts will explore data augmentation techniques to enhance model performance.

Another constraint is the retrospective nature of our system, which may not readily translate into real-time clinical decision-making. Real-time diagnosis requires immediate and accurate predictions, demanding the development of a system that can be seamlessly integrated into clinical workflows. This aspect will be a focus of our future work.

Addressing the complexities surrounding PDA diagnosis, particularly the ongoing debate over the treatment of PDA and the resultant need for a clear distinction between symptomatic and asymptomatic cases, is challenging. This decision, informed by the retrospective nature of our study, acknowledges that individual physician biases at a single center could influence PDA categorization. By dividing the groups based on treatment status, we aimed to minimize bias and reflect the clinical decision-making process more accurately. However, we recognize this approach’s limitations, as debates over PDA treatment and changing guidelines over the last decade could affect the classification of PDAs as symptomatic or incidental. This limitation underscores the importance of future research expanding data collection to multiple centers. Such expansion will not only address the bias associated with single-center studies but also allow for a broader examination of diverse PDA cases, improving the generalizability and applicability of our findings across various healthcare settings.

Despite these limitations, our experiments with medical professionals demonstrated the potential of our system in assisting PDA diagnosis. Our proposed system empowered medical professionals with comprehensive data visualization and AI-based data analysis. However, transitioning to real-time diagnosis is a complex task that requires careful consideration and technical innovation. To address these limitations and challenges, future work will involve data collection from multiple institutions to enhance the system’s adaptability to different healthcare contexts [52]. We also recognize the importance of extending the scope of the diagnostic support system for exploring the predictive ability regarding the outcomes of PDA closure. This exploration is expected to contribute significantly to providing medical professionals with the necessary tools to make informed decisions among various therapeutic interventions for newborns with PDA [53]. Lastly, we are actively exploring the creation of a real-time diagnostic support system that can effectively function in clinical settings [54]. By leveraging advanced technologies, we aim to provide timely and efficient support to medical professionals in diagnosing and managing PDA cases, ensuring that our system aligns with the dynamic and time-sensitive nature of clinical practice.

## 6. Conclusions

In this study, we introduced a comprehensive AI-based PDA diagnostic support system designed to assist medical professionals in achieving timely and accurate diagnoses in premature infants. The system combined a data viewer, data analyzer, and AI-based diagnosis supporter to provide a holistic approach to PDA diagnosis. Our evaluation revealed that the early detection models demonstrate the system’s efficacy, with notable achievements such as 84% accuracy and the ability to detect PDA symptoms up to 3.3 days in advance. This capability significantly enhanced early diagnosis, supported by feedback from medical professionals who reported improved diagnostic metrics and expressed satisfaction with the AI-based diagnosis supporter.

Building upon these promising results, our future work will focus on conducting prospective studies to validate the effectiveness of our diagnostic support system in real-time clinical settings. Additionally, we aim to expand our data collection to multiple institutions, addressing the limitations of our current single-center study and ensuring the system’s adaptability and applicability to a broader range of healthcare environments. These next steps are crucial for advancing our understanding of PDA diagnosis in premature infants and for integrating our system more seamlessly into clinical workflows, ultimately contributing to improved neonatal care.

## Figures and Tables

**Table 1:** Comparative analysis of demographic and clinical characteristics between asymptomatic and symptomatic PDA groups (SGA: small for gestational age; IUGR: intrauterine growth restriction; ^1^ Student’s *t*-test; ^2^ chi-squared test).

Characteristic	Asymptomatic PDA (*n* = 277)	Symptomatic PDA (*n* = 132)	*p* Value
Patient information			
Male gender (%)	53.07	49.24	0.536 ^2^
Gestational age (weeks)	29.82 ± 2.89	27.05 ± 1.94	<0.001 ^1^
Birth weight (grams)	1161.32 ± 272.69	953.27 ± 235.46	<0.001 ^1^
SGA (%)	42.24	21.21	<0.001 ^2^
Apgar score (1 min)	4.05 ± 1.51	3.09 ± 1.57	<0.001 ^1^
Apgar score (5 min)	6.10 ± 1.43	5.25 ± 1.74	<0.001 ^1^
Maternal medication			
Magnesium (%)	23.10	25.00	0.766 ^2^
Steroids (%)	69.31	68.94	1.000 ^2^
Maternal diagnosis			
Chorioamnionitis (%)	16.61	25.00	0.061 ^2^
Preeclampsia (%)	28.16	21.21	0.168 ^2^
Fetal anomaly			
Nervous system (%)	0.36	0.76	1.000 ^2^
Respiratory system (%)	1.44	0.76	0.913 ^2^
Cardiovascular system (%)	3.25	0.00	0.083 ^2^
Kidney (%)	0.36	0.76	1.000 ^2^
Skeletal system (%)	0.36	0.76	1.000 ^2^
Digestive system (%)	0.72	0.00	0.825 ^2^
Placenta (%)	24.19	24.24	1.000 ^2^
Other (%)	1.44	0.76	0.913 ^2^
IUGR (%)	24.91	13.64	<0.050 ^2^

**Table 2:** Categorization of extracted features for feature set (Mat: maternal; SGA: small for gestational age; Min: minimum; Max: maximum; Med: median; Avg: average; P25/P75: 25th/75th percentile; STD: standard deviation; Var: variance).

Category	Features
Prenatal (19)	Mat medication (2), Mat diagnosis (2), Echocardiography finding (9), Gender, Gestational age, Birth weight, SGA, Apgar 1/5 score
Postnatal (80)	{Min, Max, Med, Avg, P25, P75, STD, Var} of Pulse rate, Body temperature, Respiration, Systolic blood pressure (SBP), $pCO_{2}$, $pO_{2}$, Diastolic blood pressure (DBP), pH, Base excess of extracellular fluid ($BE_{ecf}$), Plasma bicarbonate concentration ($\left[HCO_{3}^{-}\right]$)
BP-related (9)	Difference between {Med} of SBP and {Med, P25, P75} of DBP, Difference between {P25} of SBP and {Med, P25, P75} of DBP, Difference between {P75} of SBP and {Med, P25, P75} of DBP

**Table 3:** Performance of PDA early detection models when using our designed feature set (FN rate: false negative rate).

Model	Feature Set			Performance			
	Prenatal	Postnatal	BP-Related	Accuracy	Precision	Recall	FN Rate
Model_prenatal	O	X	X	71%	24%	61%	39%
Model_postnatal	X	O	X	83%	39%	72%	28%
Model_perinatal	O	O	X	82%	38%	76%	24%
Model_perinatal + bp	O	O	O	84%	42%	71%	29%

## Data Availability

Data are contained within the article.

## Abbreviations

The following abbreviations are used in this manuscript:

PDA	Patent ductus arteriosus
AI	Artificial intelligence
EHR	Electronic health record
NICU	Neonatal intensive care unit
CDSS	Clinical decision support system
BP	Blood pressure
SBP	Systolic blood pressure
DBP	Diastolic blood pressure
RF	Random forest
FN	False negative
